# Role of media surveillance function during COVID-19 breakout

**DOI:** 10.1192/j.eurpsy.2021.1741

**Published:** 2021-08-13

**Authors:** A. Naveed, S. Masood

**Affiliations:** 1 -, private, karachi, Pakistan; 2 Department Of Psychology, University of Karachi, Karachi, Pakistan

## Abstract

**Introduction:**

It is a digital era and people always turn on television to gain knowledge of what’s happening around, same is the case with COVID-19 breakout. Whole world relied on media to understand the scenario, as media has always played substantial role in providing information regarding precaution and treatment of the disease.

**Objectives:**

To assess influence of media surveillance function on society during COVID-19 breakout.

**Methods:**

Participants included in the study are 11 and above who can utilize abstract thinking as per Piaget’s theory of cognitive development. Questionnaire used in the study was Media surveillance questionnaire, it was previously used in the study done during Ebola Virus Disease (EVD) spread in Nigeria. In current study the questionnaire was used by replacing disease name i.e. EVD to COVID-19. Questionnaire was distributed online on social media groups.

**Results:**

According to data analysis majority agrees that major source of COVID-19 news is television and Radio, messages on media help in avoiding sick people, media provides helpful information regarding disease prevention and control and messages on media needs more clear directions.

**Conclusions:**

Media played positive role during COVID-19 breakout and it also spread awareness about the myths, false home remedies and precautionary measures. Media also informed society about the health emergencies in case if anyone is infected with the virus. Still media messages need more clarity and direction as people rely more on media than social media. It is media’s responsibility to provide accurate information and more clear messages after inquiring matter properly especially when it’s about human life

**Disclosure:**

No significant relationships.

